# The Medical Society of London

**Published:** 1888-02

**Authors:** 


### MEDICAL SOCIETY OF LONDON.

### Meeting, December 5.

Dr. Stephen Mackenzie gave a demonstration of the Filaria Sanguinis Hominis in blood and urine, and read a paper on a case in a patient under his care. He also went very minutely into the natural history of this insect, and as to whether or not it passes through the intestinal tract of the mosquito, en route for the human lymphatics. Interesting as were these remarks they had not, of course, that novelty which gave this branch of research its exceeding interest some years since. Dr. Mackenzie has had one or two very able predecessors in this department, but this has not prevented his adding to our knowledge of the subject.

Dr. Thorowgood read a paper on "Cardiac Failure in Pulmonary Obstruction." He had obtained very good results with tartar emetic to relax the capillaries, and nux vomica to stimulate the cardiac muscle. He mentioned the case of a child freely treated by stimulants until she was livid and unconscious, while the chest was full of coarse *râles*. The administration of small doses of the vinum antimonialis with fifteen-grain doses of sulphate of magnesia, was followed by complete recovery. He expressed the opinion that no remedy could replace the tartrate of antimony to relax capillary spasms and reduce congestion when secretion was established and the skin moist; then, if cardiac failure impended, he saw that alcohol was invaluable.

Dr. Routh called attention particularly to the effects of nitro-glycerine in just such cases. He had rescued quite a number of people by giving a quantity of a one-per-centum solution of nitro-glycerine on appropriate occasions.

Dr. Habershon lauded the hypodermic injection of strychnine in cases of impending cardiac failure.

